# Supplementary material for: Identifying severe COVID-19 risk variants modulating enhancer reporter activity in lung cells
Source: PLoS Genet. 2026 Jul 17;22(7):e1012222. doi: 10.1371/journal.pgen.1012222 (PMC13399529; doi:10.1371/journal.pgen.1012222)
Supplement: Attachment — Submitted filename: 166792_1_rebuttal_3752908_ttvkz4.docx [file pgen.1012222.s015.docx]

**Manuscript number:** RC-2026-03453

**Corresponding author(s):** 1. Elias T. Friman, 2. Simon C. Biddie, 3. Wendy A. Bickmore

## 1. General Statements [optional]

We thank the reviewers for their supportive comments and thoughtful suggestions to improve our manuscript. Below we address these in a point-by-point response.

## 2. Point-by-point description of the revisions

Reviewer #1

Major comments: 
1) The study focused on regulatory activity in a lung-derived cellular setting and was well executed. However, the degree that non-coding variation in lung elements, particularly alveolar basal epithelial cells, modeled by A549 cells, contributes genetic risk for COVID19 severity is unclear. Especially as non-coding variants in other contexts such as immune cells have been shown to be enriched for disease risk. To strengthen the choice for the A549 cellular context, the authors can assess enrichment for COVID19 severity heritability using stratified LD-score regression (PMID: 26414678) using A549/lung epithelium chromatin data (ATAC-seq, CHIP-seq) to check for enrichment polygenic signal or if the lung associated-risk is focused on a restricted set of genome-wide significant signals.

The reviewer is correct that most analysis of non-coding variants to date has been in immune cells, as is the case for many GWAS studies. However, severe COVID-19 affects many systems, especially the lung alveolar epithelium, and so there is a pressing need for functional genomic studies that go beyond immune cells. We chose A549 due to its lung origin, experimental tractability, and availability of published datasets. While enrichment such as S-LDSC suggested by the reviewer would be a good indication to screen for cell types with enrichment in e.g. open chromatin, many of our STARR-seq hits were found in closed chromatin and so would have been missed by such an analysis. To further emphasize the importance of type II alveolar epithelial cells in severe COVID-19 progression, we added the following to the manuscript:  *“Cell death and the innate immune response of type II alveolar epithelial cells, which also function as progenitors for type I epithelial cells, are the main driver of alveolar damage and acute respiratory distress syndrome in coronavirus infection (Bridges et al 2022 PMID 34404754; Qian et al 2013 PMID: 23418343).”*

2) It would strengthen the manuscript to compare the results to prior analyses where overlap exists (eg PMID:36763080). Particularly it would be informative to address if nominated variants for signals have different variants operating in different cell types. Also, one prominent variant, rs17713054, that had previously been nominated to operate in lung through in silico predictions and CRISPR perturbations (PMID:34737427) appears to be non-significant in this STARR-seq analysis. Was a different variant nominated at this locus? Could the authors expand on methodological differences that could explain this difference?

rs17713054 (chr3:45,818,159:G>A) was nominated by Downes et al (2021) based on *in silico* predictions. While the authors found rs17713054 resides in open chromatin and is a chromatin accessibility (ca)QTL, the variant did not validate in CRISPR perturbations. Deletion of the putative enhancer encompassing rs17713054 across 4 cell lines led to no detectible changes in expression of the predicted target gene *LZFTL1*. The lack of H3K27ac at the putative enhancer led the authors to conclude that this enhancer is not active in any of the lung epithelial cell lines tested, consistent with our STARR-seq results which suggest that rs17713054 is inactive in A549 cells.

We nominated 6 amVars at the *LZFTL1* locus (Table 1) and propose there are multiple functional variants with small effect sizes operating at this locus which together significantly contribute to risk. We have included an additional supplemental figure panel (Fig. S2H) showing a genome browser view of these variants. As suggested by the reviewer, we also compared our results to Jagoda et al. That study only reported allele-specific change and not baseline activity, it is thus possible that very weak signal (below our thresholds) can show up as allele-specific. This appears to be the case for at least one variant (rs35454877) which we call as inactive but nevertheless has a significant allele-specific activity (mpralm padj<0.05). One of the variants they show increases activity (rs71327024) is shown to decrease activity in another study, albeit in other cell types (Uvarova et al. PMID 37762093), and has no allele-specific effect in our assay. Regarding methodological differences, we took an unbiased approach asking which variants modulate enhancer activity (in A549 cells), whereas Downes et al asked which variants are within open chromatin, considering 95 distinct cell lines, and Jagoda et al picked introgressed candidates based on an analysis pipeline and prior results in K562 cells. Furthermore, the length of the construct, position of the enhancer, and minimal promoter, readout, and analysis method, differ between the studies. We have added these comparisons to the manuscript in the first results section (see revised manuscript lines 192-201)

3) Given a subset of the prioritized variants originate from the credible set, were the amVars enriched in terms of posterior inclusion probability than the tested set? This technical information could be valuable for interpreting fine-mapping efforts.

We did not observe an enrichment of posterior inclusion probabilities (PIPs) for the amVars or active variants compared to inactive variants. One reason could be that we primarily find variants in weak enhancers with moderate effect sizes which may be too subtle to be attributed a high PIP by GWAS due to insufficient statistical power. It is also possible that variants with a high PIP are active in other cell types. Fine-mapped variant sets already contain variants likely to be functional, so observing no difference between already statistically likely functional variants is perhaps not surprising. Another study testing melanoma risk variants similarly observed no statistically significant differences in PIPs between MPRA functional and non-functional variants (Long et al 2022 PMID: 36423637). We have included a supplemental plot of the PIP scores (Fig. S2G) for inactive, active and amVars and added this analysis in the first results section (see revised manuscript lines 181-186).

4). Similarly, for the eQTL comparisons, what proportion of the amVar/eQTL pairs are directionally consistent (e.g. increased activity/increased expression)?

For the 29 amVars, there are a total of 4689 combined eQTLs across all GTEx tissues. When filtering for lung, there are 180 eQTLs across the 29 amVars, whereby only 17/29 amVars have eQTLs in lung. For 16 of these 17 amVars, there is at least 1 eQTL in lung that is directionally concordant - listed in Table 1. Notably, however, almost all variants which have lung eQTLs with concordant direction also have lung eQTLs with discordant direction, suggesting the effects may be more complicated. When considering all lung eQTLs in GTEx v11, amVars were surprisingly enriched for discordant direction of effect (see figure below, left). However, we noticed this signal was driven entirely by the variants in the H2 haplotype block (as proposed by the reviewer in question 5), which includes many genes with varying effects which may be unrelated to our amVars. When excluding chr17, no enrichment was seen (see figure below, right). There was also no significant correlation between the effect size magnitude of eQTL and STARR-seq. Therefore, globally comparing amVars and eQTLs was not informative per se. We emphasize that we have few amVars (29), which makes subtle correlations/enrichments less likely to be detectable. Siraj *et al.* (2026) (PMID: 41741648), testing a much larger variant set than ours and in multiple cell lines, observed weak correlation between MPRA allelic effects and eQTL normalized effect size (Spearman;s p = 0.35), although these libraries were selected to include only fine-mapped eQTLs in high PIP, in comparison to our libraries which also include a large number of additional variants in LD. Overall, this suggests eQTL effect size is not a strong predictor for variant effects observed by MPRAs. We have included a discussion about this (see revised manuscript lines 186-190).

5) Several of the variants implicated by STARR-seq, including several of the pairs with non-additive activity were associated with the MAPT locus. This locus has a common 900kb inversion in Europeans (PMID: 15654335), were these variants linked to the same H1/H2 haplotype?

Indeed, all five prioritised variant pairs, as well as 5 amVars and 2 further variant pairs showing STARR-seq activity at this locus (Table 1, Table 2), are linked to the same (H2) haplotype. More specifically, all variants show high LD with the H2 haplotype-tagging SNP rs8070723‐G in European ancestry (r^2^ > 0.73) and are not linked to one of the H1 haplotype-tagging SNPs (rs242557-A, r^2^ < 0.19). We have added this interesting observation to the discussion, stating *“Of note, this is a 1.8Mb region of linkage disequilibrium having two major haplotypes, and all five variant pairs as well as 5 amVars identified at this locus are linked to the H2 haplotype which encompasses a 900kb inversion (PMID17555970)”.*

6) Were the variants with non-additive effects analyzed for transcription factor motifs?

We looked for both motifs (FIMO and motifbreakR) and predictions of contribution scores using Malinois and AlphaGenome in the non-additive combinations, without finding any evidence for synergistic binding/activity. For example, see below the non-additive example in Fig. 3C (rs77819001_rs76667867), where the total activity prediction by Malinois is low (0.10-0.14), and there is no evidence of non-additive contribution scores as expected from the STARR-seq results. Because of the few examples, we cannot determine whether this is due to a systematic inability of the models to predict non-additivity, and therefore we chose not to present them. For transparency, we added the following sentence to the results: *“For prioritized, non-additive variants pairs neither model identified an impact on transcription factor motifs that could explain the observed non-additivity. However, the few examples preclude drawing any general conclusions regarding the ability of these models to detect non-additivity.”*

Minor comments: 
1) The sentence in the methods, Variant selection and design section, "the 95% credible set from the second GenOMICC releases containing causal variants to 95% statistical probability," is somewhat unclear. Given that the next sentence describes the 99% credible set, the authors should use more consistent terminology.

For the 3^rd^ release we used the 99% credible set of variants to increase comprehensiveness of our library, meaning the list of variants contains causal variants to 99% statistical probability. In contrast, for the first and second release we used the 95% credible set as is the standard for fine-mapped variants. We clarified the phrasing in the methods as follows: *“Fine-mapped severe COVID-19 risk variants encompassing causal variants to 95% statistical probability (95% credible set) from the first and second GenOMICC release^2^ and a more comprehensive 99% credible set of variants from the third GenOMICC release^2^ were included in the STARR-seq library.”*

2) Some text in supplemental figures (Fig S6) is too small to be legible. Please either remove or adjust the figure size.

We removed the variant IDs from figure panels S6A and S6B to aid readability.

Reviewer #2

Major comments:
1). The major conclusion is well supported by the main data presented; but additional clarification and extension in the discussion part may be helpful to determine the potential impacts and application of such conclusions especially related splicing isoform changes regulated by potential functional variants. "OPTIONAL" CRISPR editing for a couple of selected genes/variants will be helpful to confirm effects of these novel pathways.

We thank the reviewer for the positive appraisal.

Regarding splicing isoform changes, rs2297480 lies within the promoter-proximal region of two alternative FDPS isoforms which lack the penultimate exon encoding part of the catalytic domain. Therefore, we propose the variant could increase expression of a non-enzymatically functional FDPS isoform, which may compete with the functional isoform for substrate binding, thereby decreasing overall FDPS enzymatic activity. There are other examples of such “promoter usage” QTLs. We have rephrased this section and included references to studies supporting such a situation at other loci *“While speculative, global analyses have found examples where enhancer/promoter variants are proposed to lead to isoform expression changes (so called promoter usage QTLs), which may have disease implications (PMID 36037215, 30618377)”*

While CRISPR editing could be interesting, it would require extensive additional resources and is outside of the scope of the current manuscript. As a significant proportion of our amVars are not within accessible chromatin nor overlapping active chromatin modifications, we expect these to be functional in a different cell type rather than A549. Identifying a suitable cell line for CRISPR editing would therefore be non-trivial. Furthermore, the small effect size of our hits suggests seeing clear effects of single variants on transcription may be hard, as genes can be controlled by multiple enhancers simultaneously.

Minor comments:
1, please be specific about the proportion here in the text "Similarly, the proportion of active candidate sequences overlapping predicted ENCODE CREs in A549 cells was increased compared to inactive sequences (Fig. S2F)."

We added the specific proportions to the text: *“Similarly, the proportion of active candidate sequences (39.3%) overlapping predicted ENCODE CREs in A549 cells was increased compared to inactive sequences (23.2%) (Fig. S2E).”*

2, Out of 29 variants showing allele-specific effects, how many of them are close to the known TSS of candidate genes. Is IFNA is the only gene nearby these 29 variants?

rs7041102/rs7040981 reside ~4.5-5.5kb from the TSSs of IFNA10 and IFNA16. More gene-proximal variants include rs2297490, residing within in the first intron of the reference *FDPS* isoform and in the promoter-proximal region of three further *FDPS* isoforms (discussed above). In addition, rs145951274 resides in the first intron of *HCG27* and rs3130925 in the first intron of the reference *MICB* isoform and within the promoter region of an alternative *MICB* isoform. We have added information on the distance to the closest TSS for all 29 amVars in Table 1 and indicated whether the variant is intronic.

3, out of 166 variants, what are genes with TSS closer to the 166 variants. It will be helpful to have a table or list of these genes since their promoter close to the significant variants

Of 166 active variants, 20 are within 1kb of the nearest TSS. Of those, 16 occur in the 1kb upstream or 100bp downstream of the TSS, the other 4 variants are within 1kb downstream. We have added this information as a new column to Supplementary Table 3 now showing the distance to the nearest TSS for all single variants tested, and we have modified figure S2F (previously S2E) which now shows the comparison of TSS proximity for inactive, active, and amVars.

4, Do these 16 combinations of variants pairs have genetic interaction in the population levels, i.e. epistasis?

This is an intriguing point, but challenging to test and beyond the scope of this work. 12/16 variant pairs are in very high or perfect LD (Table 2) and therefore either both risk variants or neither will co-occur in the population. We therefore cannot test if two variants have epistatic (beyond additive) effects in the population, nor can we directly link individual variants to a biological phenotype and therefore test for epistatic effects on phenotypes. We are limited to testing for beyond additive, i.e. epistatic, regulatory effects in the context of the STARR-seq assay, which we show in Fig 3B.

5, It needs more clarification why the risk allele of rs2297480 at the FDPS locus is associated with increased enhancer activity and decreased levels or activity of FDPS?

We have addressed this point under major comment 1 in the context of enhancer/promoter-driven isoform switches as plausible disease mechanism.

To clarify, the rs2297480 risk allele showed increased enhancer activity by STARR-seq. The variant lies within the promoter-proximal region of two alternative FDPS isoforms which lack the penultimate exon encoding part of the catalytic domain. Therefore, we propose the variant could increase expression of a non-enzymatically functional FDPS isoform, thereby decreasing overall FDPS enzymatic activity as the enzymatically inert isoform may compete with the functional isoform for substrate binding. We emphasize that this possible mechanism at FDPS is speculative.
